# Long-term lifestyle monitoring adherence in patients after cardiac intervention: a prospective observational trial

**DOI:** 10.1093/ehjdh/ztag016

**Published:** 2026-01-30

**Authors:** Wilhelmina Francisca Goevaerts, Nicole Catharina Christina Wilhelmina Tenbült - Van Limpt, Sebastiaan André Goossen, Max Valentin Birk, Yunjie Liu, Marta Regis, Rutger Willem Maurice Brouwers, Yuan Lu, Willem Johan Kop, Hareld Marijn Clemens Kemps

**Affiliations:** Department of Industrial Design, Eindhoven University of Technology, PO Box 513, 5600 MB, Eindhoven, The Netherlands; Department of Cardiology, Máxima Medical Center (Eindhoven/Veldhoven), PO Box 7777, 5500 MB, Veldhoven, The Netherlands; Department of Industrial Design, Eindhoven University of Technology, PO Box 513, 5600 MB, Eindhoven, The Netherlands; Department of Cardiology, Máxima Medical Center (Eindhoven/Veldhoven), PO Box 7777, 5500 MB, Veldhoven, The Netherlands; Department of Industrial Design, Eindhoven University of Technology, PO Box 513, 5600 MB, Eindhoven, The Netherlands; Department of Industrial Engineering and Innovation Sciences, Eindhoven University of Technology, Eindhoven, The Netherlands; Department of Industrial Design, Eindhoven University of Technology, PO Box 513, 5600 MB, Eindhoven, The Netherlands; Department of Mathematics and Computer Science, Eindhoven University of Technology, Eindhoven, The Netherlands; Department of Research and Medical Innovation, Máxima Medical Center, Eindhoven/Veldhoven, The Netherlands; Department of Industrial Design, Eindhoven University of Technology, PO Box 513, 5600 MB, Eindhoven, The Netherlands; Department of Cardiology, Máxima Medical Center (Eindhoven/Veldhoven), PO Box 7777, 5500 MB, Veldhoven, The Netherlands; Department of Industrial Design, Eindhoven University of Technology, PO Box 513, 5600 MB, Eindhoven, The Netherlands; Department of Medical and Clinical Psychology, Center of Research on Psychological Disorders and Somatic Diseases, Tilburg University, Tilburg, The Netherlands; Department of Industrial Design, Eindhoven University of Technology, PO Box 513, 5600 MB, Eindhoven, The Netherlands; Department of Cardiology, Máxima Medical Center (Eindhoven/Veldhoven), PO Box 7777, 5500 MB, Veldhoven, The Netherlands

**Keywords:** Cardiac rehabilitation, Wearable electronic devices, Self-monitoring, Adherence, Usability, Health behaviour change

## Abstract

**Aims:**

Lifestyle behaviours are important predictors of morbidity and mortality in patients with cardiovascular disease. However, structured lifestyle monitoring is insufficiently integrated into clinical practice. This study evaluated dropout and long-term adherence to an eHealth system for self-monitoring lifestyle behaviours in patients with cardiovascular disease.

**Methods and results:**

Patients undergoing a cardiac intervention used an eHealth system (web application with integrated health watch and chatbot) to monitor physical activity, nutrition, stress, and sleep for 1 year. The primary outcome was dropout, defined as system disengagement. Secondary outcomes included adherence (percentage of prescribed health watch wear time and chatbot responses) and usability. The predictive value of demographic, clinical, and psychosocial factors was examined using logistic regression models. Of 100 patients (mean age 61.6 ± 10.4 years; 88% male; 45% percutaneous coronary intervention, 55% other intervention), there were 43 dropouts; most (27; 63%) occurred in the first quarter, with participation burden being the most cited reason (51%). Health watch adherence was higher than chatbot adherence (80.7% (66.6–90.3%) vs. 60.8% (30.7–82.7%), *P* < 0.001). Low chatbot adherence was associated with poorer mental well-being, while lower health watch adherence was associated with higher levels of depressive symptoms. System usability was rated acceptably usable (62.2 ± 14.7).

**Conclusion:**

Long-term lifestyle monitoring of multiple health-related behaviours is feasible after cardiac intervention, highlighting its potential for integration into clinical practice. Patient engagement could be enhanced by targeting subgroups at risk of low adherence, particularly in the early phase, and by reducing self-reporting burden while improving usability.

## Graphical Abstract

**Graphical Abstract:**
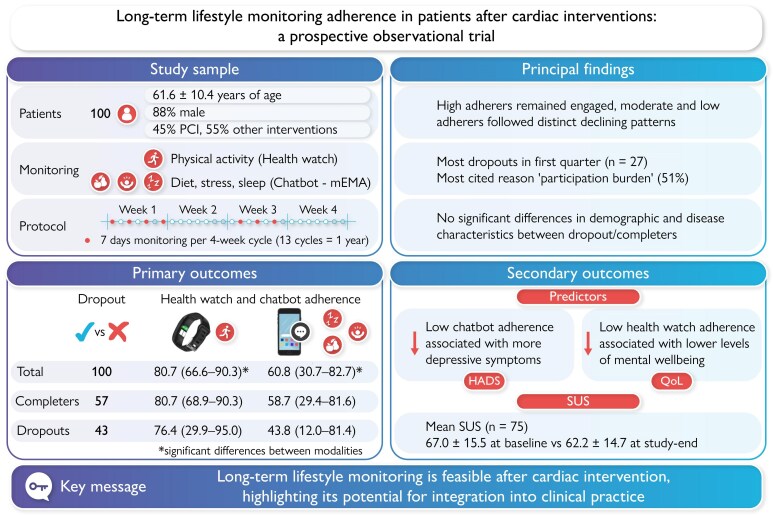
HADS, Hospital Anxiety and Depression Scale; mEMA, mobile Ecological Momentary Assessment; PCI, Percutaneous Coronary Intervention; QoL, Quality of Life; SUS, System Usability Scale. Values are presented as mean (± SD) for normally distributed variables and as median (IQR) for non-normally distributed variables.

## Introduction

Cardiovascular disease (CVD) remains the leading cause of morbidity and mortality worldwide,^1^ placing a significant burden on healthcare systems and patients’ quality of life.^2^ Lifestyle behaviours, such as physical inactivity, poor dietary habits, mental stress, and poor sleep quality, play a crucial role in influencing cardiovascular risk and outcomes by worsening the clinical course of cardiac diseases.^3^ Lifestyle interventions reduce the risk of recurrent cardiac events and complications in patients with chronic cardiovascular conditions, such as coronary artery disease (CAD) and atrial fibrillation (AF). Cardiorespiratory fitness also predicts the occurrence of arrhythmias in patients with CAD^4^ and AF,^5^ highlighting the beneficial effects of increasing physical activity to improve fitness. Improving lifestyle behaviour is a key component of cardiac rehabilitation (CR) programmes. Despite the evidence supporting the advantages of multimodal CR, the majority of patients who have undergone cardiac interventions continue to maintain unhealthy lifestyles,^6^ for example, sedentary behaviour and obesity due to unhealthy dietary habits, which are major cardiovascular risk factors. These findings indicate that current cardiac rehabilitation programmes are not sufficiently used or effective in achieving long-term behavioural change. To support sustainable lifestyle behaviour change, it is essential that both patients and healthcare professionals gain deeper insights, through objective feedback, into the patients’ lifestyle behaviour changes over time. This will enable more informed decision-making and facilitate tailored interventions that promote long-term success.

Lifestyle behaviours are quantified using a broad range of measures, ranging from objective measures like physical activity (e.g. step count) to subjective measures like perceived stress, making it challenging to identify a single, clear method for assessing these behaviours. Yet, advances in technology have enhanced both the accuracy and accessibility of these measurements. For instance, algorithms combining heart rate and accelerometry measures provide more precise data on physical activity than step counts alone,^7^ and innovations in the tools for monitoring nutritional intake, supported by increasingly detailed food composition databases, make dietary tracking more feasible and accesible.^8^ In addition, the growing advances and confidence in mobile ecological momentary assessments (mEMA) for capturing subjective data,^9^ such as stress and sleep, are paving the way for more reliable and convenient methods leading to reproducible, long-term self-monitoring of lifestyle behaviour.

As digital technologies become more accessible, monitoring lifestyle behaviours is becoming less burdensome and more feasible. Innovations such as activity trackers enable unobtrusive measurement of physical activity, and the widespread use of smartphones ensures that data entry is user-friendly and easily accessible, potentially improving adherence. Furthermore, by providing continuous feedback and encouraging regular engagement, these technologies offer opportunities for individuals to reflect on and adjust their behaviours. Behaviour change theories emphasize the importance of self-regulation as a key determinant of behaviour change. Self-monitoring is considered one of the strategies that supports self-regulation by increasing one’s awareness, which can, in turn, foster empowerment and facilitate behaviour change.^10^ Therefore, lifestyle assessment and tracking through self-monitoring tools hold the potential to initiate and sustain behaviour change. However, long-term self-reporting of lifestyle habits often faces issues related to adherence,^11^ indicating the need to explore adherence patterns and identify strategies that support sustained engagement and improve outcomes over time.

To investigate patterns of dropout, adherence, and potential predictors of long-term lifestyle monitoring with a custom-built integrated eHealth system, a prospective observational trial was conducted. The eHealth monitoring system combines wearable sensors, a chatbot, and a dashboard for self-monitoring lifestyle behaviours in patients with CVD.^12^ By combining multiple methods to measure lifestyle behaviours, the system provides a comprehensive combination of both objective metrics and subjective lifestyle factors, offering a holistic approach to self-monitoring in CVD care. The primary purpose of this trial was to investigate dropout and non-adherence and to identify their potential predictors in patients who underwent a cardiac intervention. The insights from this study serve to guide the integration of structured lifestyle monitoring into routine care, ultimately enhancing cardiovascular health outcomes.

## Methods

### Study design and setting

The study design of the CardiOVascular Research Opting for New Applications (Care-On) trial is described in detail elsewhere.^12^ In brief, the Care-On clinical trial is a 1-year prospective observational trial evaluating dropout, adherence, predictors of dropout and adherence, and usability.

The trial protocol was reviewed and approved by the institutional review board of the Máxima Medical Center, and the clinical trial was registered in the Dutch Trial Register (registration number: NL9861). All patients provided written informed consent before study entry. The Care-On clinical trial was conducted according to the Declaration of Helsinki. The results are described according to the RECORD^13^ reporting guidelines, which extend the STROBE statement, with the corresponding checklist included in Supplementary material online, *Table S1*.

### Patient selection and procedures

Patients referred for or recently having undergone an invasive cardiac intervention were considered for participation in this trial. Eligible interventions were: coronary artery bypass surgery (CABG), a (fractional flow reserve [FFR]-guided) percutaneous coronary intervention (PCI), radiofrequency catheter ablation (RFCA), valve surgery including transcatheter aortic valve implantation (TAVI) (see *Table 1* for relevant patient characteristics). In cases where only a diagnostic procedure (i.e. FFR) was performed and no intervention was deemed necessary, patients were still included because of the initial treatment intent and their comparable clinical context. A total of 103 patients were initially enrolled in the study. Of these, three were excluded because of the following reasons: change in treatment regimen resulting in no longer meeting the inclusion criteria (*n* = 2) and death before the intervention (*n* = 1). The patients were asked to self-monitor their lifestyle for one year after discharge, using the Care-On platform with smartphone application and integrated health watch, measuring physical activity, dietary habits, alcohol use, psychological stress, and sleep quality.

**Table 1:** Baseline characteristics of the sample (completers vs. dropouts)

	Total sample (*n* = 100)	Completers (*n* = 57)	Dropouts (*n* = 43)	OR (95% CI)	*P-*value
Age, years	61.6 ± 10.4	62.5 ± 9.9	60.3 ± 11.0	1.02 (0.98–1.06)	0.28
Male, *n* (%)	88 (88%)	50 (88%)	38 (88%)	0.94 (0.28–3.19)	0.92
Primary cardiac diagnosis					
Myocardial infarction	30 (30%)	17 (30%)	13 (30%)	0.98 (0.41–2.32)	0.97
Atrial fibrillation	9 (9%)	6 (11%)	3 (7%)	1.57 (0.37–6.66)	0.54
Atrial flutter	3 (3%)	2 (4%)	1 (2%)	1.53 (0.13–17.42)	0.73
Angina pectoris	27 (27%)	14 (25%)	13 (30%)	0.75 (0.31–1.83)	0.53
Dyspnoea	19 (19%)	12 (21%)	7 (16%)	1.37 (0.49–3.84)	0.55
Ventricular arrhythmia	1 (1%)	1 (2%)	0 (0%)	N/A	N/A
No symptoms	12 (12%)	6 (11%)	6 (14%)	0.73 (0.22–2.43)	0.60
Primary cardiac intervention or procedure					
CABG	25 (25%)	15 (26%)	10 (23%)	1.18 (0.47–2.96)	0.73
PCI	45 (45%)	25 (44%)	20 (47%)	0.90 (0.41–1.99)	0.79
FFR	3 (3%)	1 (2%)	2 (5%)	0.37 (0.03–4.18)	0.42
RFCA	13 (13%)	9 (16%)	4 (9%)	1.83 (0.52–6.39)	0.35
Valve surgery	19 (19%)	12 (21%)	7 (16%)	1.37 (0.49–3.84)	0.55
Other	1 (1%)	1 (2%)	0 (0%)	N/A	N/A
LVEF ≤ 35%, *n* (%)	0 (0%)	0 (0%)	0 (0%)	N/A	N/A
BMI, kg/m^2^	27.6 ± 4.1	27.2 ± 3.9	28.2 ± 4.2	0.94 (0.85–1.04)	0.24
Currently smoking	12 (9%)	4 (7%)	8 (19%)	0.33 (0.09–1.18)	0.09
Attended cardiac rehabilitation modules					
Physiotherapy	73 (73%)	43 (75%)	30 (70%)	1.33 (0.55–3.23)	0.53
Dietician	29 (29%)	19 (33%)	10 (23%)	1.65 (0.67–4.04)	0.27
Medical psychologist	18 (18%)	10 (18%)	8 (19%)	0.93 (0.33–2.60)	0.89
Sociodemographic measures					
Currently employed, *n* (%)	55 (55%)	32 (56%)	23 (53%)	0.90 (0.41–1.99)	0.79
Higher education, *n* (%)	30 (30%)	19 (33%)	11 (26%)	0.69 (0.29–1.66)	0.40
Marital status					
Married or domestic partnership	82 (82%)	48 (84%)	34 (79%)	Ref	0.52
Single, never married	8 (8%)	5 (9%)	3 (7%)	0.85 (0.19–3.79)	0.83
Divorced or widowed	10 (10%)	4 (7%)	6 (14%)	0.40 (0.06–2.70)	0.35

BMI, body mass index; CABG, Coronary Artery Bypass Graft; FFR, Fractional Flow Reserve; LVEF, left ventricular ejection fraction; OR, odds ratio; PCI, Percutaneous Coronary Intervention; RFCA, radiofrequency catheter ablation.

Values are presented as mean (±SD) for continuous variables and as *n* (%) for categorical variables. For continuous predictors, an OR < 1 indicates that higher values of the variable are associated with higher odds of dropout. For categorical predictors, an OR < 1 suggests that belonging to the specified category is associated with higher odds of dropout compared to the reference category. N/A: Odds ratio not estimable because of low count or separation issues. Ref: Reference variable. Due to small sample sizes in diagnosis and intervention groups, results should be interpreted with caution.

Patients were also asked to complete a standardized lifestyle behaviour assessment via the platform at baseline, 3, 6, 9, and 12 months. These assessments also included validated questionnaires on quality of life and usability. Additional psychosocial factors were assessed using supplementary questionnaires during the baseline assessment. All self-report measures were administered in Dutch via the lifestyle monitoring platform (see below for details).

Furthermore, sociodemographic measures (i.e. marital status, employment status, and level of education) were obtained during the baseline visit. Cardiovascular risk factors (i.e. age, sex, smoking status, body mass index [BMI]) and clinical characteristics (i.e. primary CVD diagnosis, left ventricular ejection fraction, and intervention type) were obtained from the electronic patient records (EPRs).

### Study equipment

The lifestyle monitoring system includes four main features: a health watch, a chatbot, a personal dashboard, and a goal-tracking module. Detailed information about the system is described elsewhere.^12^ In brief, the health watch was used to collect objective data on physical activity (e.g. step count, activity type) and related physiological parameters (e.g. heart rate, energy expenditure, cardio fitness index). The chatbot was used to gather self-report data using mEMAs for sleep (based on the Consensus Sleep Diary^14^), nutrition (using the 24-h dietary recall method with an integrated dietary database^15^ based on the Dutch Food Composition Database [NEVO]^16^), and mental stress (based on the Positive Affect Negative Affect Schedule [PANAS]^17^). Patients could also track their measures, adherence, and goals (optional), and share data with practitioners and relatives (optional).

The mEMA schedule followed a recurring 4-week cycle, consisting of two active reporting weeks and two off weeks. During the active weeks (Week 1 and Week 3 of each cycle), patients received five prompts per day on selected days. In Week 1, prompts were delivered on Monday, Wednesday, Friday, and Sunday. In Week 3, prompts were delivered on Tuesday, Thursday, and Saturday. This structure resulted in a total of seven reporting days and 35 prompts per 4-week cycle. No prompts were sent during Week 2 and Week 4, allowing for alternating periods of reporting and non-reporting. Patients were also asked to wear the health watch for at least 12 h per day during the active weeks.

The monitoring system was implemented as an unguided intervention. Following the initial onboarding session, participants engaged with the system independently, without further contact with healthcare professionals. Data collected via the platform were presented to participants through visual summaries to facilitate self-monitoring. Physical activity, sleep, and stress data were displayed in graphical formats, while nutritional intake was shown in a food diary with an accompanying traffic light system (green, yellow, red) to indicate the relative healthiness of individual food items. Additionally, automated lifestyle advice was generated based on responses to the periodic lifestyle assessments. No personalized coaching or professional interpretation of the data was provided during the study period.

### Outcomes

The primary outcome of the clinical trial was dropout from the trial. Secondary outcomes were adherence to the study protocol (i.e. answering the chatbot prompts and wearing the health watch), changes in lifestyle over time, and perceived usability of the lifestyle monitoring system.

#### Dropout status

Patients were classified as dropouts when they decided to quit, or no activity was registered across any of the study components (chatbot, health watch, and quarterly questionnaires via the dashboard) for a period of at least 2 months. Patients were reminded after 1 month of no activity; after another month of no activity, they were classified as dropouts. This definition aligns with the primary outcome described in the protocol, which focused on the proportion of participants who completed the 1-year follow-up. In the current article, we also report usage-based adherence in more detail to capture patterns of engagement throughout the study.

#### Adherence

Adherence was assessed based on patients’ engagement with the two primary study components: wearing the health watch for at least 12 h per day during measurement weeks and responding to chatbot prompts (i.e. responding to 35 prompts spread over 7 days within a 4-week period [i.e. one cycle]). Percentages were calculated for each of these components to evaluate overall adherence to the study protocol in total and per group. For the ‘completers’-group (i.e. the group of patients having completed the full study year), adherence was calculated as the percentage of required wearing time and the percentage of chatbot prompts for the full study year. For patients classified as dropouts, adherence data were included up to the last week of active participation, defined as the final week in which data were provided for any module (i.e. chatbot or watch). If a patient decided to quit after a buffer week that coincided with the start of a new quarter, any data from that buffer week was excluded from both the adherence calculation for the succeeding quarter and the calculation of weeks of active engagement. This approach was chosen to ensure consistency across all patients, avoid skewing adherence rates by prolonged ‘0’ adherence values, and focus solely on meaningful engagement during active participation periods, excluding incidental or minimal data provided during non-mandatory buffer weeks.

#### Psychosocial predictors of dropout and adherence

Several baseline psychosocial constructs were assessed as potential predictors of dropout and adherence, of which the selection was based on previous literature. Following the review of Leung *et al*.^18^ ‘stage of change and motivation’, ‘self-efficacy’, ‘anxiety and depressive symptoms’, ‘general levels of perceived stress’, and ‘quality of life’ (i.e. physical and mental well-being), were included as potential predictors of adherence. Furthermore, fatigue and self-reported physical fitness were included because fatigue and reduced physical fitness are frequently reported symptoms during the course of CVD, and play a key role in prognosis and quality of life.^19^ Mobile device proficiency and perception of system usability were also included, given their influence in the acceptance and continued use of digital applications.^20^ Since self-reported physical fitness, general levels of perceived stress, quality of life, and system usability are also analysed as secondary study outcomes, they are described in detail in their respective sections. The following validated instruments were used for the psychosocial constructs:

- *Stage of change and motivation:* The Readiness-to-Change Lifestyle (RTCLQ) and Confidence-to-Change Lifestyle (CTCLQ) Questionnaire,^21^ based on the Transtheoretical Model of Behaviour Change.
- *Self-efficacy:* The General Self-Efficacy Scale (GSES).^22^
- *Anxiety and depressive symptoms:* The Hospital Anxiety and Depression Scale (HADS).^23^
- *Fatigue:* The Fatigue Assessment Scale (FAS).^24^
- *Technology proficiency:* The Mobile Device Proficiency Questionnaire (MDPQ-16).^25^

#### Lifestyle behaviours and quality of life during the 1-year care-on trial

Lifestyle behaviour (i.e. physical fitness, mental stress, dietary habits, alcohol intake, smoking status, sleep quality, and medication adherence) and quality of life questionnaires were administered at baseline, 3, 6, 9, and 12 months. The following validated instruments were used:

- *Physical fitness:* The FitMáx-questionnaire^26^ for estimating cardiorespiratory fitness (VO_2_peak).
- *General levels of perceived stress:* The Perceived Stress Scale (PSS-10).^27^
- *Diet quality and alcohol intake:* The NutriMáx Questionnaire, which provides standardized scores for overall diet quality and alcohol separately (see example of the LifeStyleScore-study.^28^)
- *Sleep quality:* The Pittsburgh Sleep Quality Index (PSQI).^29^
- *Tobacco smoking:* A single-item smoking status question (yes/no).
- *Medication adherence:* The THORESCI-trial medication adherence questionnaire.^30^
- *Quality of life and well-being:* The Short Form Health Survey (SF-12),^31^ which measures physical (PCS-12) and mental (MCS-12) well-being aspects of quality of life.

#### Perception of system usability

The System Usability Scale (SUS)^32^ was administered at baseline and at study completion or dropout to be able to assess differences in perception of usability over time. The SUS is a 10-item questionnaire scored from 0 to 100, where higher scores indicate better usability. A score of 68 is considered average, while scores above 80 reflect excellent usability and below 60 suggest usability concerns.

### Statistical analysis and sample size

Descriptive statistics were provided for demographic and baseline characteristics. Normally distributed variables were summarized as mean ± SD with independent *t*-tests for comparing dropout and completers; non-normally distributed variables were summarized as median (interquartile range [IQR]) and compared via the Mann–Whitney *U*-test. Categorical variables were presented as *n* (%).

The association between the primary outcome (dropout) and demographic, clinical, and psychosocial variables was assessed using logistic regression models. Initially, univariate analyses were conducted, reporting odds ratios (ORs) with 95% confidence intervals (CIs) and *P*-values (where OR < 1 indicates higher odds of dropout). Stepwise logistic regression was then performed in three stages: (1) age and sex, (2) clinical variables, and (3) psychosocial factors. A similar model was run in which baseline physical fitness and quality of life were included in the third stage instead of psychosocial factors, to account for multicollinearity. Only the stepwise logistic regression analysis adjusted for demographic and clinical variables, while further analyses focused solely on dropout or adherence.

For the analysis of adherence, Wilcoxon signed-rank test was used for comparing health watch and chatbot adherence. Spearman’s correlation assessed the relationship between chatbot and health watch adherence, while chi-square tests examined dropout chance across quarters and differences between adherence groups (>75%, 50–75%, <50%). Adherence trends between groups were analysed by reporting means with 95% CIs, and group differences were assessed using the Kruskal-Wallis H test with post-hoc pairwise comparisons. Logistic regression evaluated whether early adherence predicted dropout.

Longitudinal outcomes (i.e. lifestyle changes and SUS scores) were analysed using linear mixed models (LMMs) to account for repeated measurements. Fixed effects included time, group variables (dropout status or adherence group), and their interactions. A first-order autoregressive (AR1) covariance structure was chosen after performing model selection based on the AIC, considering the data characteristics and consistency within the study. Models were estimated using restricted maximum likelihood), and estimated marginal means were reported for group comparisons for SUS scores.

To assess whether SUS scores predicted dropout, binary logistic regression was conducted, grouping patients by SUS categories (>68 vs. ≤ 68), with ORs and 95% CIs reported. All analyses were performed using SPSS (version 29.0, IBM Corp.).

The sample size calculation was based on the primary outcome (dropout status). A sample of 100 patients allowed for detecting a 50% dropout rate over one year with a precision of ±10% (95% CI: 0.40–0.60).

#### Data access, linkage, and cleaning methods

Clinical data were retrieved from EPRs, platform usage (including chatbot prompt response rate and health watch wear time) was collected via system logs, and patient-reported outcomes were obtained through questionnaires administered via the platform. Each patient was assigned a unique study identifier, which was used to link platform usage logs, clinical data from the EPRs, and questionnaire answers. All identifiable information was removed after linkage to maintain privacy. Linkage was performed on encrypted datasets stored on a secure server with access limited to authorized researchers.

## Results

### Study sample

Of the 100 patients included in the study (mean age 61.6 ± 10.4 years, median 62.0 years (range: 39.0–81.0); 88% male; 45% PCI, 55% other intervention), 43 were classified as dropout. The baseline characteristics of the total sample and the two groups separately (completers vs. dropouts) are described in *Table 1*. No significant differences were found between the groups of completers and dropouts on the clinical and demographic measures presented in *Table 1*.

### Dropout

**Figure 1:**
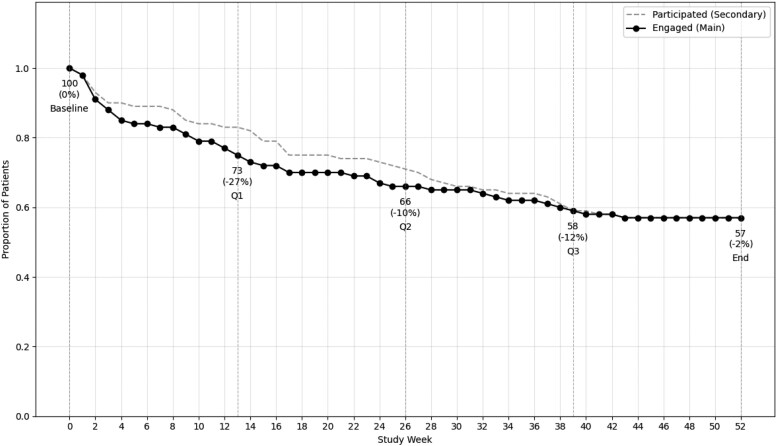
Proportion of patients participating in the study and actively engaging with the platform over time. The dashed grey line represents the proportion of patients who were still enrolled in the study each week (‘Participated’), regardless of whether they provided any data. The solid black line shows the proportion of patients who actively provided data through the health watch or chatbot that week (‘Engaged’). Labels beneath the data points show the number of patients engaged at each time point, along with the percentage change in disengagement from the previous time point, calculated relative to the remaining patients.

*Figure 1* illustrates patient engagement and participation over time. The number of weeks engaged represents the number of weeks that the participant actively engaged with the platform, as described in the ‘Methods’ section. The number of weeks participated is the actual number of weeks that the patients were in the study, e.g. before having decided to quit, or being classified as dropout. Among dropouts, the mean duration of engagement was 13.8 ± 13.1 weeks (median: 11 weeks), while the mean duration of study participation was 17.6 ± 13.4 weeks (median: 16 weeks). Of the 43 dropouts, 27 patients (−27%, disengagement relative to remaining patients) disengaged in the first quarter, seven (−10%) during the second quarter, eight (−12%) in the third quarter, and one (−2%) in the fourth quarter. Dropout rates differed significantly between quarters (*P* < 0.001). Reasons for dropout included high participation burden (*n* = 22, 51%) and personal reasons (*n* = 19, 44%), with one patient withdrawing for undisclosed reasons (2%) and one because of death (2%). High participation burden included the following aspects: technical problems (synchronization issues or ergonomic/malfunction-related dissatisfaction with the health watch) and self-report burden (dissatisfaction with the burden or perceived inefficiency of the chatbot).

### Adherence

*Table 2* presents adherence percentages per quarter and for the full study year. Over the 12-month study period, health watch adherence was significantly higher than chatbot adherence (80.7 (66.6–90.3) vs. 60.8 (30.7–82.7), *P* < 0.001). Median health watch adherence was slightly lower for dropouts compared to completers (76.4 (29.9–95.0) vs. 80.7 (68.9–90.3)) and, similarly, median chatbot adherence was slightly lower for dropouts compared with completers (43.8 (12.0–81.4) vs. 58.7 (29.4–81.6)), but these differences were not statistically significant (*P* = 0.22 and *P* = 0.11, respectively).

**Table 2:** Percentage adherence from baseline to 12 months and adherence subgroup distribution

Module	Q1	Q2	Q3	Q4	Study year	Low adherers	Moderate adherers	High adherers
Adherence to chatbot (%)								
Total (100)	64.0 (43.5–86.8)	62.9 (30.7–84.9)	61.6 (25.2–80.8)	54.4 (20.0–76.9)	60.8 (30.7–82.7)	45% (*n* = 45)	23% (*n* = 23)	32% (*n* = 32)
Completers (57)	63.9 (41.3–87.0)	62.9 (30.5–83.9)	61.4 (23.8–80.8)	54.2 (19.2–76.9)	58.7 (29.4–81.6)	35% (*n* = 20)	33% (*n* = 19)	32% (*n* = 18)
Dropouts (43)	44.8 (12.0–84.0) (*n* = 43)	61.9 (24.5–85.5) (*n* = 16)	38.6 (24.9–78.0) (*n* = 9)	46.6 (*n* = ­1)	43.8 (12.0–81.4)	58% (*n* = 25)	9% (*n* = 4)	33% (*n* = 14)
Adherence to health watch (%)								
Total (100)	89.8 (70.8–99.3)	89.6 (68.9–99.0)	86.2 (65.8–96.4)	75.4 (40.1–95.0)	80.7 (66.6–90.3)	21% (*n* = 21)	17% (*n* = 17)	62% (*n* = 62)
Completers (57)	90.3 (71.1–99.4)	89.6 (67.9–99.0)	86.2 (63.3–96.4)	76.6 (40.5–95.0)	80.7 (68.9–90.3)	11% (*n* = 6)	19.3% (*n* = 11)	70% (*n* = 40)
Dropouts (43)	77.8 (33.0–98.3) (*n* = 43)	71.8 (8.3–94.6) (*n* = 16)	79.2 (31.7–89.8) (*n* = 9)	73.3 (*n* = ­1)	76.4 (29.9–95.0)	35% (*n* = 15)	14.0% (*n* = 6)	51% (*n* = 22)

Values are presented as median (IQR). Values for dropouts represent adherence up to their last recorded participation.

Overall, a weak-to-moderate positive correlation was found between health watch and chatbot adherence (*ρ* = 0.395, *P* < 0.001), indicating that higher health watch adherence was associated with higher chatbot adherence. Given this association, adherence was also analysed separately in three subgroups for illustrative purposes: high adherers (>75% adherence), moderate adherers (50–75% adherence), and low adherers (<50% adherence). In addition to the adherence percentages, *Table 2* presents the distribution of the three subgroups within the total sample, as well as among completers and dropouts.

#### Adherence trends

**Figure 2:**
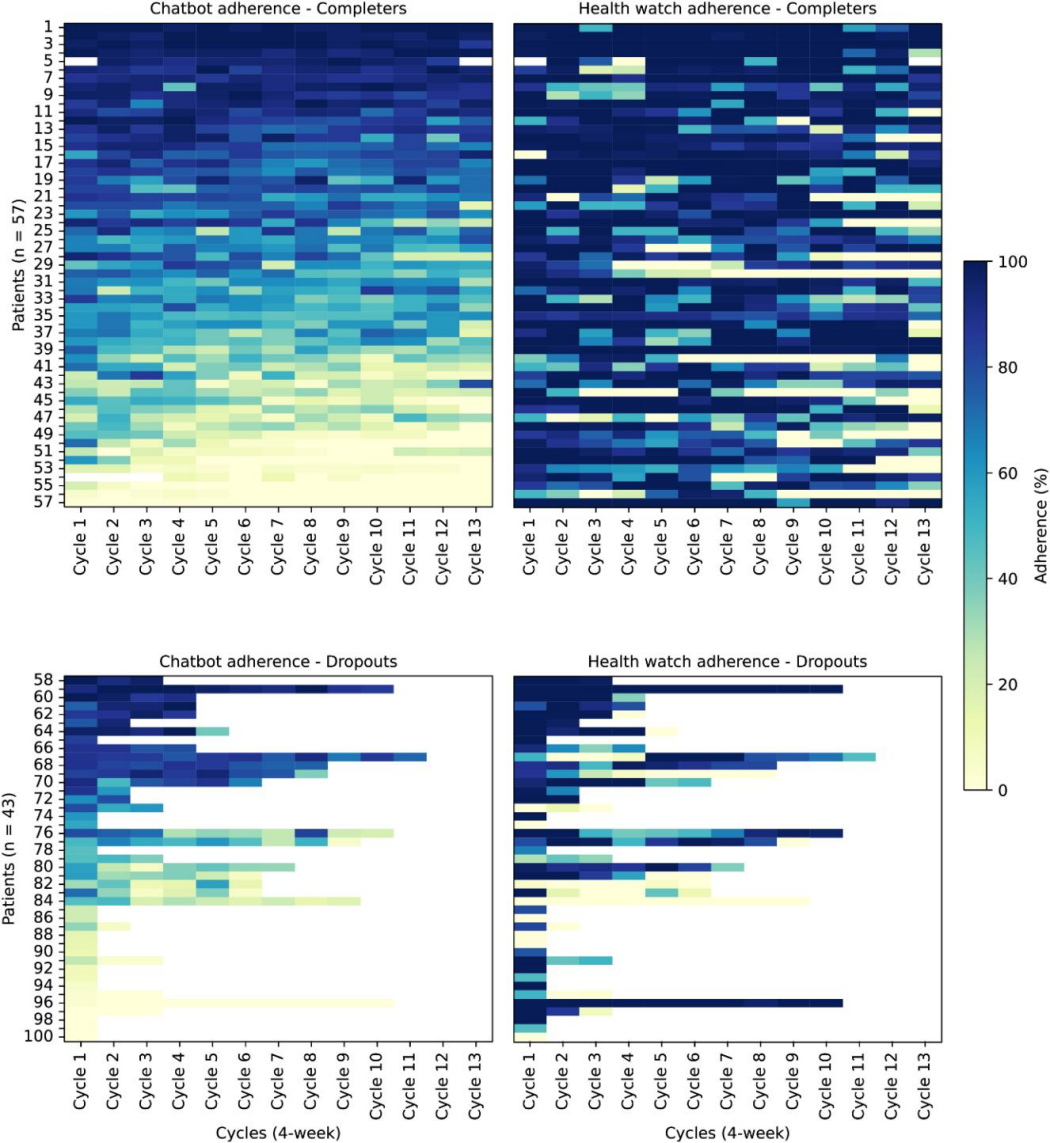
Percent adherence per patient across 4-week cycles from baseline to 12 months displayed as heatmaps. Patients are sorted based on the average chatbot adherence within groups, and the health watch adherence heatmaps are aligned accordingly. Darker colours indicate higher adherence.

Adherence percentages were calculated for each 4-week cycle, separately for health watch wear time and chatbot response compliance, and visualized for completers and dropouts in *Figure 2*. Trends were examined across the three adherence groups over the full year.

Adherence rates for both the chatbot and the health watch exhibited a general decline over time, with distinct patterns among low, moderate, and high adherence groups (*P* < 0.001). High adherers consistently demonstrated higher adherence compared to both moderate and low adherers (*P* < 0.001), while moderate adherers maintained significantly higher adherence levels than low adherers in the majority of cycles (*P* < 0.05). High chatbot adherers remained stable throughout the study, starting at 89.4% in Cycle 1 and slightly declining to 87.4% by Cycle 13. Moderate chatbot adherers began with relatively high adherence (70.9%) and gradually decreased to 47.7% by Cycle 13. Low chatbot adherers started at 31.8% and dropped sharply to 14.9% by Cycle 13, with the steepest decline occurring in the early cycles. Similarly, high health watch adherers maintained stable adherence from 92.3% in Cycle 1% to 65.4% by Cycle 13. Moderate adherers started at 86.1% and declined to 49.0% by the final cycle, showing a more gradual decrease compared to the chatbot. Low health watch adherers began with 43.2% and dropped to 0% by Cycle 11, maintaining this level until the study end.

A logistic regression model revealed that early adherence (i.e. the adherence in the first 4 weeks [Cycle 1] to both the chatbot (model fit: *P* = 0.01) and health watch (model fit: *P* = 0.01) significantly predicted dropout risk. For chatbot adherence, the OR was 0.98 (95% CI: 0.97–1.00, *P* = 0.01), indicating that lower adherence in the first cycle increased the likelihood of dropout. Similarly, for health watch adherence, the OR was 0.98 (95% CI: 0.96–1.00, *P* = 0.01), showing that lower early adherence with the health watch was associated with a higher dropout risk. Of the initial sample, three completers were excluded from the regression analysis due to missing data caused by data handling discrepancies (non-patient-related): two for chatbot data (one for the first and last cycles, the other for the first three cycles) and one for health band data during the first cycle.

### Psychosocial predictors of dropout and adherence

In *Table 3*, the outcomes of the baseline psychosocial measurements and their relation to dropout are displayed. Ten patients (10%) were not analysed because they did not complete the baseline questionnaires. No significant differences were found between groups in terms of state of change, self-efficacy, and psychological factors (see the respective sections for the association between lifestyle factors and quality of life measures, as well as usability factors, with dropout and adherence).

**Table 3:** Baseline outcomes of psychosocial measurements of the sample (completers vs. dropouts)

Variable	Instrument	Scoring range; interpretation	Total sample (*n* = 90)	Completers (*n* = 56)	Dropouts (*n* = 34)	OR (95% CI)	*P-*value
Psychosocial factors							
Stage of Change	Readiness-to-Change Lifestyle Questionnaire	1–5; (1) maintenance—(5) contemplation	2.5 ± 1.4	2.5 ± 1.3	2.5 ± 1.4	0.99 (0.72–1.35)	0.93
Confidence to Change	Confidence-to-Change Lifestyle Questionnaire	1–3; (1) not very confident—(2) somewhat confident—(3) very confident	2.4 ± 0.5	2.5 ± 0.5	2.4 ± 0.6	1.33 (0.60–2.93)	0.49
Self-efficacy	General Self-Efficacy Scale (GSES)	10–40; higher = greater self-efficacy	32.2 ± 5.1	32.6 ± 4.4	31.6 ± 6.0	1.04 (0.96–1.13)	0.35
Anxiety	Hospital Anxiety and Depression Scale (HADS)	0–21; higher = more anxiety/more depressive symptoms	3.0 (1.0–7.0)	3.0 (1.0–7.0)	3.0 (1.0–7.0)	0.97 (0.88–1.06)	0.52
Depressive symptoms	Hospital Anxiety and Depression Scale (HADS)	0–21; higher = more anxiety/more depressive symptoms	3.0 (1.0–7.0)	3.0 (1.0–5.5)	3.0 (1.0–7.0)	0.97 (0.88–1.07)	0.59
Fatigue	Fatigue Assessment Scale (FAS)	10–50; higher = more fatigue	22.0 ± 8.0	21.5 ± 7.0	23.0 ± 9.5	0.98 (0.93–1.03)	0.37
Mobile Device Proficiency	Mobile Device Proficiency Questionnaire (MDPQ-16)	8–40; higher = greater proficiency	36.3 (31.0–39.0)	36.8 (32.3–39.5)	36.0 (29.0–39.0)	1.05 (0.99–1.12)	0.09

CI, confidence interval; IQR, interquartile range; OR, odds ratio.

Values are presented as mean (±SD) for normally distributed variables and as median (IQR) for non-normally distributed variables. An OR < 1 suggests that higher values of the predictor variable are associated with higher odds of dropout, whereas an OR > 1 indicates lower odds of dropout.

#### Multivariate analysis

Logistic regression analysis was used to examine predictors of dropout status while avoiding multicollinearity among the psychological variables (see Supplementary material online, *Table S2*). Age and sex were initially included, but did not significantly predict dropout. Additional clinical variables (i.e. cardiac diagnosis, intervention, and BMI) were tested but were not significantly associated with dropout status. Similarly, none of the psychosocial variables were significant predictors of dropout.

Psychosocial variables were also analysed as potential predictors of adherence group (low, moderate, and high adherence). Logistic regression analyses were performed, combining the high (>75%) and moderate (50–75%) adherence groups and comparing them to the low adherence group (<50%), as adherence above 50% was considered sufficient for analysing habitual lifestyle patterns. For health watch adherence, higher depressive symptoms were associated with low adherence (OR 0.87, 95% CI 0.77–0.99, *P* = 0.03), suggesting a role of psychological well-being in device usage. No significant associations were found for the chatbot adherence groups.

### Lifestyle behaviour and quality of life

**Figure 3:**
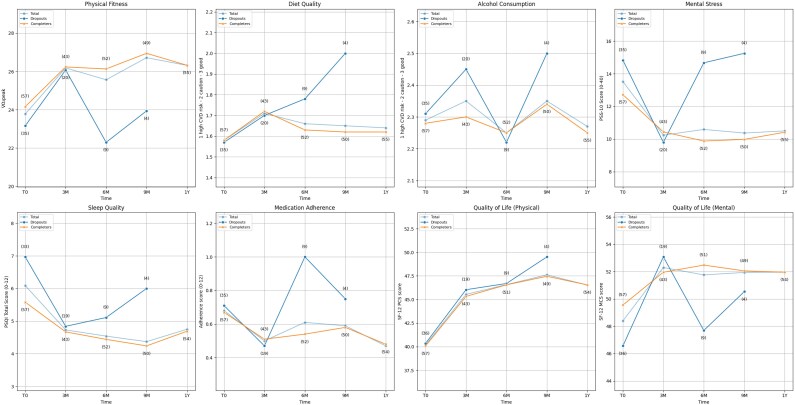
Change over time for lifestyle behaviours and quality of life. Results are presented separately for completers, dropouts, and the total sample. Measurements were taken at baseline (T0), 3 months (3 M), 6 months (6 M), 9 months (9 M), and 12 months (1Y, study end). The annotations (#) above the data points indicate the number of patients who provided data at each time point for that group. Outcomes and scoring: physical fitness (V̇O₂peak), diet quality and alcohol consumption (1: high cardiovascular risk behaviour, 2: caution, 3: good), mental stress (0–40; higher = greater perceived stress), sleep quality (0–12; higher score = worse sleep quality), medication adherence (0–12; higher score = worse adherence), quality of life (mental and physical components score; 0–100: >50 better than average, <50 below average).

*Figure 3* summarizes changes in lifestyle behaviours, that is, physical fitness, diet quality, alcohol use, smoking status, stress, sleep, medication adherence, and quality of life over time, that is, baseline, 3, 6, 9, and 12 months, for the total sample and by group (completers vs. dropouts). Dropouts were asked to fill out a final questionnaire upon withdrawal, with data assigned to the nearest quarter for comparability. As dropout responses declined over time, that is, only four in Q3, none at study end, group comparisons should be interpreted with caution. As smoking status remained largely unchanged throughout the study, further analyses were only performed for the other lifestyle behaviours and quality of life.

Baseline physical fitness and quality of life were examined as additional potential predictors of dropout and adherence. A similar stepwise approach was used for the psychosocial measurements. Baseline data for lifestyle and quality of life measures were missing for eight patients (8%). Better mental well-being (i.e. a higher SF-12 MCS score) was associated with higher chatbot adherence (OR 1.07, 95% CI: 1.02–1.12, *P* = 0.01), while no significant associations were found for health watch adherence or dropout status.

Over the study period, improvements were observed in physical fitness (*P* = 0.04), diet quality (*P* = 0.01), sleep quality (*P* < 0.001), and reductions in mental stress (*P* = 0.002). Both physical (SF-12 PCS, *P* < 0.001) and mental (SF-12 MCS, *P* = 0.01) quality of life and well-being improved over time. Dropouts exhibited greater variability in their outcomes compared to completers, which may be attributed to the smaller sample size and individual differences within this group. Overall, trends were similar across adherence groups, with no clear differences observed based on chatbot or health watch use.

### Perception of system usability

The analysis of SUS scores at baseline vs. study-end included 75 patients (54 completers, 21 dropouts). The end-of-study questionnaire was not completed by 22 dropouts (for whom it was optional) and 3 completers (because of illness [*n* = 2], undisclosed [*n* = 1]). SUS scores were significantly lower at the end of study than at baseline (67.0 ± 15.5 [baseline] vs. 62.2 ± 14.7, *P* = 0.004), with a greater decline for dropouts (62.8 ± 2.5 [baseline] vs. 54.2 ± 3.0) than for completers (66.3 ± 2.0 [baseline] vs. 64.2 ± 2.0; *P* = 0.08), indicating decreased perceived usability among dropouts. While baseline SUS scores were not predictive of dropout risk (OR 1.01, 95% CI: 0.97–1.04, *P* = 0.32), patients with lower usability (SUS < 68) at study-end were more likely to have dropped out (OR 0.34, 95% CI: 0.11–1.05, *P* = 0.06), but this association did not reach statistical significance. Given that a SUS score of 68 is considered the average usability threshold, the overall mean score of 62.2 ± 14.7 at study end indicates marginally acceptable usability, suggesting the system was usable but with noticeable usability limitations.

### Discussion

This study evaluated a newly designed holistic lifestyle monitoring system aimed at promoting long-term self-monitoring in patients with CVD. The system was used by more than half of the patients for a year following a cardiac intervention, indicating that long-term lifestyle self-monitoring is a feasible option as an integral part of chronic cardiac care. The majority of dropouts occurred in the first quarter, with participation burden being the most frequently cited reason. Low chatbot adherence was associated with lower mental well-being, whereas low health watch adherence was associated with more depressive symptoms. Significant improvements in lifestyle behaviours were observed over time, independent of adherence and dropout. The system’s usability is in the acceptable range, indicating room for improvement, with lower usability scores among dropouts. These findings emphasize the need for strategies to reduce the participation burden, enhance usability, improve digital health readiness, and support engagement in digital self-monitoring interventions, particularly in subgroups such as patients with lower mental well-being or depression.

#### Early dropout: critical window for dropout prediction

Sustained engagement with digital health systems is essential for achieving meaningful health outcomes, yet dropout remains a persistent barrier, as also demonstrated in this study. The first quarter, the initial study phase, proved to be a critical phase, with most dropouts occurring early, a common pattern in digital health interventions,^33,34^ where the first few weeks or months often predict long-term retention. Additionally, lower adherence in the first two quarters was associated with higher dropout rates, particularly for the health watch, emphasizing that early engagement strategies are essential to sustain participation over time. Early reinforcement mechanisms, such as personalized onboarding, for example, early structured support, tutorials, and real-time feedback, can enhance initial engagement and should, therefore, be considered when implementing lifestyle self-monitoring in clinical practice.^35^

#### Distinct adherence patterns

Engagement patterns varied significantly between adherence groups. While high adherers remained engaged, moderate and low adherers followed distinct declining patterns. Low chatbot adherers disengaged more rapidly than low watch adherers, indicating that active self-reporting may be more challenging to sustain than passive monitoring, which has also been documented in prior studies.^36^ Additionally, while both chatbot and health watch adherence declined over time, chatbot adherence showed a steeper initial drop, whereas health watch adherence remained relatively stable before declining in later phases of the trial. Tailored engagement strategies need to be adapted to each system component, ensuring that both self-reporting and continuous monitoring remain sustainable over time. Self-reporting requires active effort, potentially making it harder to sustain than passive monitoring, and requires a balance of motivation, ability, and triggers.^36^ By recognizing these differences, digital interventions can implement engagement strategies that account for the varying demands of different tracking modalities.

#### Overcoming technical and practical barriers

Participation burden and usability challenges were the most frequently cited reasons for dropout, highlighting the need for systems that are both effective and easy to use. Persistent technical issues, such as connectivity failures, charging problems, and usability concerns, may have discouraged continued participation, reducing the motivation and perceived feasibility of sustained use. This aligns with research showing that perceived ease of use is a major determinant of technology adoption, and usability perceptions directly influence engagement, as explained by the Technology Acceptance Model.^37^ This can explain why early watch adherence was a significant predictor of study completion, as there were more technical difficulties with the health watch early on in the study. Patients with higher mobile device proficiency may have been better equipped to troubleshoot issues, which could explain why it showed a stable effect across models in predicting engagement.

#### Psychosocial factors: individual differences in engagement

Although psychosocial features were not predictive of dropout, higher levels of depressive symptoms were associated with lower health watch adherence and lower mental well-being with reduced chatbot adherence, reinforcing the role of psychological health in digital health engagement. For chronic disease populations, who often face physical, emotional, and cognitive challenges, maintaining long-term adherence can be particularly demanding.^38^ These findings highlight the need for eHealth systems that balance usability and burden while providing valuable insights and minimizing the demands placed on users. Without this balance, digital health systems may fail to engage those who need them the most, reducing their long-term impact on patient outcomes.

#### Behaviour change: support of self-monitoring

For those who adhered to the system, the structured approach to self-monitoring likely supported sustained behaviour change by fostering self-awareness and accountability, although this was not directly observed in the results. The provision of feedback and the ability to track progress may have reinforced positive behaviours, consistent with behaviour change theories.^10^ The system’s limited ability to retain all patients suggests that its potential impact on behaviour change could be greater if adherence rates were improved. At the same time, engagement may naturally decline as behaviours become more habitual and intrinsically motivated, reflecting successful internalization rather than disengagement.^39,40^ Nonetheless, patterns of long-term adherence remain valuable, both for understanding system use and for informing potential clinical or behaviour change applications. Many patients also received intensive medical follow-up and cardiac rehabilitation, which likely supported engagement and may have complemented the effects of self-monitoring. Since the system was not paired with a targeted behavioural intervention, its full potential to drive change remains unexplored, and it is possible that integrating structured support could further enhance outcomes. Long-term effects may also take time to manifest, as lifestyle behaviours often deteriorate over time.^6^ Future research could examine how structured self-monitoring supports behaviour change across different patient populations and identify which strategies are most effective for sustaining engagement when needed.

### Limitations

A potential limitation of this study is the role of selection bias: individuals who choose to participate may already have been more motivated to engage in health-related interventions compared to the broader population of patients with CVD. Additionally, patients in this study may exhibit greater technological savviness, as they were not deterred by the information provided about the eHealth tools and study requirements during recruitment. This bias could result in higher adherence rates and more favourable outcomes within the study sample, limiting the generalizability of the findings to less motivated or less technologically adept patients. However, despite potential bias, the study included a relatively large and diverse sample (*n* = 100) of patients following cardiac intervention, which increases robustness of the findings. Moreover, adherence patterns varied considerably within the sample, suggesting that different levels of engagement were still captured. Importantly, even within a population that may have been initially motivated, adherence challenges were observed, reinforcing the relevance of addressing engagement barriers in real-world settings. Moreover, engagement with eHealth in chronic disease management, including cardiac care, is increasing^41^ as telemonitoring becomes more widely accepted and integrated into clinical practice. While implementation has been slow, the COVID-19 pandemic accelerated adoption, and ongoing advancements in technology, digital literacy, and patient familiarity are likely to reduce selection bias in future research. Future research should explore strategies to further enhance the representativeness of study populations, ensuring inclusivity across varying levels of motivation and technological proficiency, and assess their impact on adherence and outcomes.

While the primary sample size was calculated to estimate dropout rates with ±10% precision, the study also included sufficient power to detect moderate associations in regression analyses. However, as the study is observational in nature, the associations between adherence and mental well-being should still be interpreted with caution. Causal conclusions cannot be drawn, and unmeasured confounders or bidirectional effects may play a role. We consider these findings to be hypothesis-generating and encourage future confirmatory research.

Another limitation of this study was the incomplete data on technical difficulties, which may have led to an underestimation of adherence rates. Frequent synchronization issues with the health watch were reported by the patients to the researchers in the early phases of the clinical trial, potentially affecting adherence measurements. Some patients may have engaged with the device, but due to data transmission failures, their adherence was not accurately recorded. Only a few missed chatbot prompts due to app errors or connectivity issues, which could only be distinguished from intentional non-use through patient reports.

Another consideration is the limited involvement of patients in the development phase of the monitoring system. While clinicians and one patient with CVD provided input during development, the system was not co-designed with a broader group of end users. The absence of iterative patient feedback may have affected usability and engagement, and could have contributed to some of the observed adherence challenges.

While the results of this study demonstrate the potential of the holistic approach for lifestyle monitoring in a patient group with several CVDs, further research is needed to assess its applicability across different clinical settings. Variations in healthcare environments and patient needs may influence the system’s effectiveness and engagement. Exploring its use in other healthcare contexts will help determine how well it can be integrated into routine care for other chronic conditions and whether it can be scaled up for broader implementation. Such extensions would maximize its potential to improve health outcomes in a variety of clinical settings.

#### Future implications

Despite adherence challenges, this study demonstrates that sustained use of an integrated system for self-monitoring is feasible and beneficial for long-term engagement in healthy lifestyle behaviours. Patients are willing to incorporate both active self-reporting and passive monitoring into their routines, enhancing adherence and providing a more comprehensive picture of lifestyle behaviours and health outcomes. Evidence suggests that self-guided eHealth interventions can be as effective as those supplemented by professional support, reinforcing the potential of standalone digital health solutions.^42^ However, other studies have shown that incorporating some degree of human support, such as therapist guidance or coaching, can further enhance engagement and efficacy, particularly in mental health interventions.^43^ This suggest that while fully automated systems hold promise for scalable delivery, hybrid models that combine digital self-guidance with human input may yield optimal outcomes, especially for users requiring additional motivational or emotional support. Building on these insights, the next step is to leverage the rich, longitudinal data captured by monitoring systems to deliver automated, personalized lifestyle guidance. Emerging technologies, such as large language models, could harness these data to tailor interventions more effectively to individual needs, aligning with evidence-based behaviour change techniques. To maximize impact, future interventions should minimize participation burden, address technical barriers, and offer adaptive, user-tailored support that sustains motivation and usability over time.

#### Clinical relevance

Structured monitoring can provide real-time insights into patients’ behaviours, allowing for timely interventions and personalized care plans. Additionally, it could be highly useful for determining the residual risk in patients with CVD, serving as a basis for treatment strategies. This approach not only complements traditional clinical treatments but also empowers patients to take a more active role in their own care, fostering a sense of ownership over their health. Integrating such systems into routine CVD care could lead to more consistent monitoring for risk prediction, better adherence to lifestyle recommendations, and, ultimately, a reduction in cardiovascular morbidity. Additionally, a system that aids patients in monitoring their lifestyle factors supports better self-management and boosts self-motivation, with subsequent positive effects on the lifestyle behaviours themselves. By providing personalized feedback based on collected data, eHealth tools can facilitate patients in managing their health and sustaining lifestyle behaviour changes.^10,44^

## Conclusions

The results demonstrate that a comprehensive lifestyle monitoring system can effectively support long-term self-monitoring of lifestyle behaviours in patients after a cardiac intervention, as a majority of patients remained engaged throughout the study. However, our results also showed that patient adherence and engagement can be further optimized by reducing the burden of self-reporting, addressing technical challenges, enhancing usability and engagement support in the early phase. This system holds significant promise in facilitating self-management and supporting long-term behaviour change in cardiac patients, with the potential to personalize cardiac care based on individual needs. This study also underscores the broader role of eHealth technologies in cardiovascular disease management, offering scalable solutions for continuous lifestyle monitoring and digital interventions. However, further research is needed to refine these innovations, ensuring their effectiveness, usability, and applicability in real-world settings. By addressing these challenges, digital health interventions can better support patients in adopting and maintaining healthier lifestyles, ultimately contributing to improved cardiovascular outcomes. The long-term success of these systems will depend on accessibility, sustained user engagement, and seamless integration into daily routines.

## Supplementary Material

ztag016_Supplementary_Data

## Data Availability

The data underlying this article cannot be shared publicly due to the inclusion of sensitive information. The data will be shared on reasonable request to the corresponding author.
