# Supplementary material for: Long-term lifestyle monitoring adherence in patients after cardiac intervention: a prospective observational trial
Source: Eur Heart J Digit Health. 2026 Jan 30;7(2):ztag016. doi: 10.1093/ehjdh/ztag016 (PMC12930194; doi:10.1093/ehjdh/ztag016)
Supplement: ztag016_Supplementary_Data [file ztag016_supplementary_data.zip › Supplementary Material Table ST2.docx]

**Supplementary Material Table ST2** Correlation matrix

| **Variable** | **Age** | **Sex** | **Diagnosis** | **Intervention** | **BMI** | **Stage of Change** | **Confidence to Change** | **Self-efficacy** | **Anxiety** | **Depressive symptoms** | **PSS-10** | **QoL PCS-12** | **QoL MCS-12** | **Fatigue** | **Physical fitness** | **MDPQ** | **SUS** |
| --- | --- | --- | --- | --- | --- | --- | --- | --- | --- | --- | --- | --- | --- | --- | --- | --- | --- |
| **Age** | 1.00 |  |  |  |  |  |  |  |  |  |  |  |  |  |  |  |  |
| **Sex** | -0.07 | 1.00 |  |  |  |  |  |  |  |  |  |  |  |  |  |  |  |
| **Diagnosis** | 0.06 | 0.00 | 1.00 |  |  |  |  |  |  |  |  |  |  |  |  |  |  |
| **Intervention** | 0.28^**^ | -0.01 | 0.04 | 1.00 |  |  |  |  |  |  |  |  |  |  |  |  |  |
| **BMI** | -0.29^**^ | -0.18 | -0.17 | -0.14 | 1.00 |  |  |  |  |  |  |  |  |  |  |  |  |
| **Stage of Change** | -0.20 | 0.12 | 0.12 | -0.04 | 0.05 | 1.00 |  |  |  |  |  |  |  |  |  |  |  |
| **Confidence to Change** | 0.16 | -0.02 | -0.03 | 0.00 | -0.08 | -0.43^**^ | 1.00 |  |  |  |  |  |  |  |  |  |  |
| **Self-efficacy** | 0.08 | 0.10 | -0.08 | 0.03 | -0.04 | -0.16 | 0.31^**^ | 1.00 |  |  |  |  |  |  |  |  |  |
| **Anxiety** | -0.25^*^ | -0.18 | 0.05 | -0.09 | 0.06 | 0.19 | -0.17 | -0.55^**^ | 1.00 |  |  |  |  |  |  |  |  |
| **Depressive symptoms** | -0.14 | -0.04 | 0.06 | -0.06 | 0.04 | 0.24^*^ | -0.19 | -0.58^**^ | **0.75^**^** | 1.00 |  |  |  |  |  |  |  |
| **PSS-10** | -0.27^*^ | -0.16 | -0.01 | -0.06 | 0.13 | 0.26^*^ | -0.21^*^ | -0.55^**^ | **0.81^**^** | **0.76^**^** | 1.00 |  |  |  |  |  |  |
| **QoL PCS-12** | 0.11 | 0.18 | 0.08 | 0.00 | -0.24^*^ | -0.16 | 0.10 | 0.35^**^ | -0.31^**^ | -0.45^**^ | -0.30^**^ | 1.00 |  |  |  |  |  |
| **QoL MCS-12** | 0.29^**^ | 0.01 | -0.03 | 0.09 | -0.19 | -0.21^*^ | 0.20 | 0.53^**^ | **-0.78^**^** | **-0.78^**^** | **-0.81^**^** | 0.39^**^ | 1.00 |  |  |  |  |
| **Fatigue** | -0.15 | -0.29^**^ | 0.01 | 0.03 | 0.15 | 0.16 | -0.23^*^ | -0.53^**^ | 0.67^**^ | **0.76^**^** | **0.71^**^** | -0.50^**^ | **-0.77^**^** | 1.00 |  |  |  |
| **Physical fitness** | -0.16 | 0.37^**^ | 0.02 | 0.11 | -0.29^**^ | -0.15 | 0.13 | 0.20 | -0.24^*^ | -0.34^**^ | -.29^**^ | 0.47^**^ | 0.32^**^ | -0.50^**^ | 1.00 |  |  |
| **MDPQ** | -0.16 | 0.13 | -0.10 | -0.10 | 0.08 | 0.10 | -0.05 | 0.32^**^ | -0.06 | -0.22^*^ | -0.17 | 0.12 | 0.17 | -0.24^*^ | 0.29^**^ | 1.00 |  |
| **SUS** | -0.11 | 0.03 | -0.14 | 0.12 | -0.01 | 0.03 | 0.11 | 0.18 | -0.11 | -0.14 | -0.08 | 0.19 | 0.11 | -0.07 | 0.15 | 0.33^**^ | 1.00 |

**Footnote:** ** Correlation is significant at the 0.01 level (2-tailed); * Correlation is significant at the 0.05 level (2-tailed).
